# Supplementary material for: Consensus molecular subtype differences linking colon adenocarcinoma and obesity revealed by a cohort transcriptomic analysis
Source: PLoS One. 2022 May 13;17(5):e0268436. doi: 10.1371/journal.pone.0268436 (PMC9106217; doi:10.1371/journal.pone.0268436)
Supplement: S1 Table — (DOCX) [file pone.0268436.s001.docx]

Supplemental Table 1. Patient Demographics and Tumor Characteristics by CMS Category

|  | CMS1  n = 37 | | CMS2  n = 54 | | CMS3  n = 31 | | CMS4  n = 86 | | Unassigned  n = 23 | |  |
| --- | --- | --- | --- | --- | --- | --- | --- | --- | --- | --- | --- |
|  | n | % | n | % | n | % | n | % | n | % | P-value |
| *Sex** |  |  |  |  |  |  |  |  |  |  | 0.684 |
| Female | 16 | 15 | 26 | 24 | 18 | 17 | 37 | 34 | 11 | 10 |  |
| Male | 21 | 17 | 28 | 23 | 13 | 11 | 49 | 40 | 12 | 10 |  |
| *Age** |  |  |  |  |  |  |  |  |  |  | 0.742 |
| 30-39 | 3 | 33 | 3 | 33 | 1 | 11 | 2 | 22 | 0 | 0 |  |
| 40-49 | 3 | 12 | 5 | 20 | 5 | 20 | 10 | 40 | 2 | 8 |  |
| 50-59 | 2 | 4 | 12 | 27 | 4 | 9 | 22 | 49 | 5 | 11 |  |
| 60-69 | 10 | 16 | 16 | 26 | 10 | 16 | 20 | 32 | 6 | 10 |  |
| 70-79 | 12 | 20 | 13 | 22 | 8 | 13 | 21 | 35 | 6 | 10 |  |
| 80-89 | 7 | 25 | 5 | 18 | 3 | 11 | 10 | 36 | 3 | 11 |  |
| 90> | 0 | 0 | 0 | 0 | 0 | 0 | 1 | 50 | 1 | 50 |  |
| *Ethnicity** |  |  |  |  |  |  |  |  |  |  | 0.205 |
| Hispanic/Latino | 0 | 0 | 2 | 29 | 0 | 57 | 4 | 57 | 1 | 14 |  |
| Not Hispanic/Latino | 0 | 0 | 1 | 33 | 0 | 67 | 2 | 67 | 5 | 0 |  |
| Not Reported | 37 | 17 | 51 | 23 | 31 | 36 | 80 | 36 | 22 | 10 |  |
| *Race** |  |  |  |  |  |  |  |  |  |  | 0.871 |
| American Indian/Alaska Native | 0 | 0 | 0 | 0 | 0 | 0 | 1 | 100 | 0 | 0 |  |
| Asian | 2 | 25 | 3 | 38 | 0 | 0 | 2 | 25 | 1 | 12 |  |
| Black/African American | 4 | 8 | 12 | 24 | 11 | 22 | 15 | 29 | 9 | 18 |  |
| White | 31 | 18 | 39 | 23 | 20 | 12 | 68 | 40 | 13 | 8 |  |
| *Geographic site of patients** |  |  |  |  |  |  |  |  |  |  | 0.483 |
| Non-United States | 8 | 24 | 7 | 21 | 4 | 12 | 13 | 39 | 1 | 3 |  |
| United States | 29 | 15 | 47 | 24 | 27 | 14 | 73 | 37 | 22 | 11 |  |
| *Tumor Location** |  |  |  |  |  |  |  |  |  |  | **0.004** |
| Ascending Colon | 10 | 24 | 12 | 29 | 4 | 10 | 11 | 27 | 4 | 10 |  |
| Cecum | 12 | 21 | 5 | 9 | 10 | 18 | 19 | 33 | 11 | 19 |  |
| Descending Colon | 1 | 8 | 3 | 25 | 4 | 33 | 4 | 33 | 0 | 0 |  |
| Hepatic Flexure | 3 | 21 | 5 | 36 | 2 | 14 | 4 | 29 | 0 | 0 |  |
| Rectosigmoid Junction | 0 | 0 | 1 | 100 | 0 | 0 | 0 | 0 | 0 | 0 |  |
| Sigmoid Colon | 2 | 3 | 20 | 32 | 6 | 10 | 32 | 52 | 2 | 3 |  |
| Splenic Flexure | 0 | 0 | 1 | 20 | 1 | 20 | 3 | 60 | 0 | 0 |  |
| Transverse Colon | 5 | 22 | 3 | 13 | 3 | 13 | 8 | 35 | 4 | 17 |  |
| Not Reported | 4 | 25 | 4 | 25 | 1 | 6 | 5 | 31 | 2 | 12 |  |
| *Tumor Stage** |  |  |  |  |  |  |  |  |  |  | 0.131 |
| Stage I | 8 | 24 | 9 | 27 | 7 | 21 | 7 | 21 | 2 | 6 |  |
| Stage II | 20 | 21 | 21 | 22 | 12 | 13 | 32 | 34 | 9 | 10 |  |
| Stage III | 8 | 10 | 17 | 22 | 10 | 13 | 33 | 42 | 11 | 14 |  |
| Stage IV | 1 | 4 | 7 | 28 | 2 | 8 | 14 | 56 | 1 | 4 |  |
| *Lymph Node Ratio** |  |  |  |  |  |  |  |  |  |  | 0.204 |
| LNR0 | 25 | 20 | 31 | 25 | 19 | 15 | 37 | 30 | 11 | 9 |  |
| LNR1 | 7 | 12 | 12 | 21 | 9 | 16 | 26 | 46 | 3 | 5 |  |
| LNR2 | 1 | 5 | 3 | 16 | 3 | 16 | 8 | 42 | 4 | 21 |  |
| LNR3 | 1 | 10 | 3 | 30 | 0 | 0 | 4 | 40 | 2 | 20 |  |
| LNR4 | 0 | 0 | 2 | 18 | 0 | 0 | 8 | 73 | 1 | 9 |  |
| Not Reported | 3 | 27 | 3 | 27 | 0 | 0 | 3 | 27 | 2 | 18 |  |
| *BMI**^†^ |  |  |  |  |  |  |  |  |  |  | **0.040** |
| Normal | 17 | 22 | 20 | 26 | 3 | 4 | 30 | 39 | 7 | 9 |  |
| Overweight | 9 | 12 | 12 | 16 | 16 | 22 | 30 | 41 | 7 | 9 |  |
| Obese | 11 | 14 | 22 | 28 | 12 | 15 | 26 | 32 | 9 | 11 |  |

* Significance across score categories by Fishers Exact test

^†^ BMI 19-24.9 (Normal), BMI 25-29.9 (Overweight), BMI ≥30 (Obese)
