# Supplementary material for: Consensus molecular subtype differences linking colon adenocarcinoma and obesity revealed by a cohort transcriptomic analysis
Source: PLoS One. 2022 May 13;17(5):e0268436. doi: 10.1371/journal.pone.0268436 (PMC9106217; doi:10.1371/journal.pone.0268436)
Supplement: S2 Table — normal, obese vs. overweight, and overweight vs. normal comparisons for each CMS category. (DOCX) [file pone.0268436.s002.docx]

Supplemental Table 2. Hub gene analysis for the obese vs. normal, obese vs. overweight, and overweight vs. normal comparisons for each CMS category.

|  | **hgnc_symbol** | **log2FC*** | **Padj^∇^** | **description** |
| --- | --- | --- | --- | --- |
| Obese vs. Normal^†^ | | |  |  |
| CMS1 | BSN | 2.19 | 0.007 | Bassoon presynaptic cytomatrix protein |
|  | SYP | 1.72 | 0.022 | Major synaptic vesicle protein p38 |
|  | UNC13A | 2.71 | 0.000 | Unc-13 homolog A |
|  | RAB3C | 2.99 | 0.003 | RAB3C, member RAS oncogene family |
|  | SYN2 | -2.42 | 0.025 | Synapsin II |
|  | CPLX2 | 4.70 | 0.000 | Complexin II |
|  | KIF1A | 4.63 | 0.000 | kinesin family member 1A |
|  | SYNPR | -4.44 | 0.005 | Synaptoporin |
|  | CYP1A1 | -5.24 | 0.035 | Cytochrome P450, family 1, subfamily A, polypeptide 1 |
|  | HSD17B1 | 1.02 | 0.020 | Short chain dehydrogenase/reductase family 28C member 1 |
|  |  |  |  |  |
| CMS2 | DNMT3A | -5.63 | 0.000 | DNA (cytosine-5-)-methyltransferase 3 alpha |
|  | APOA2 | 4.78 | 0.003 | Apolipoprotein A-II |
|  | ZIC2 | 3.24 | 0.046 | Zinc finger protein of the cerebellum 2 |
|  | CYP1A1 | 5.15 | 0.017 | Cytochrome P450, family 1, subfamily A, polypeptide 1 |
|  | ALDH1A2 | -3.95 | 0.001 | Retinaldehyde-specific dehydrogenase type 2 |
|  | UGT2B10 | -4.19 | 0.001 | UDP glucuronosyltransferase 2 family, polypeptide B10 |
|  | GPC3 | -2.09 | 0.039 | Intestinal protein OCI-5 |
|  | WNT6 | -3.05 | 0.004 | Wingless-type MMTV integration site family, member 6 |
|  | NTS | -4.28 | 0.003 | Neurotensin/neuromedin N |
|  | TWIST1 | 1.46 | 0.046 | Class A basic helix-loop-helix protein 38 |
|  |  |  |  |  |
| CMS3 | MAGEA6 | 23.82 | 0.000 | Melanoma-associated antigen 6 |
|  | MAGEA3 | 24.08 | 0.000 | Melanoma-associated antigen 3 |
|  | MAGEA12 | 20.72 | 0.000 | Melanoma-associated antigen 12 |
|  | MAGEA11 | 22.69 | 0.000 | Melanoma-associated antigen 11 |
|  | HOXC13 | 16.61 | 0.000 | Homeobox protein Hox-C13 |
|  | IRX2 | 7.35 | 0.012 | Iroquois-class homeodomain protein IRX-2 |
|  | TENM2 | -3.86 | 0.008 | Teneurin transmembrane protein 2 |
|  | MT4 | 17.54 | 0.000 | Metallothionein 4 |
|  | TAS2R30 | 15.83 | 0.000 | Taste 2 receptor member 30 |
|  | KRT24 | 15.90 | 0.000 | Keratin 24 |
|  |  |  |  |  |
| CMS4 | UGT1A6 | -1.84 | 0.002 | UDP glucuronosyltransferase 1 family, polypeptide A6 |
|  | UGT1A8 | -2.21 | 0.003 | UDP glucuronosyltransferase 1 family, polypeptide A8 |
|  | CYP2C9 | -2.14 | 0.011 | Cytochrome P450, family 2, subfamily C, polypeptide 9 |
|  | UGT1A1 | -3.26 | 0.000 | UDP glucuronosyltransferase 1 family, polypeptide A1 |
|  | CYP1A1 | -3.71 | 0.006 | Cytochrome P450, family 1, subfamily A, polypeptide 1 |
|  | NR1H4 | -2.79 | 0.003 | Nuclear receptor subfamily 1, group H, member 4; Isoform 4 |
|  | UGT1A10 | -1.93 | 0.002 | UDP glucuronosyltransferase 1 family, polypeptide A10 |
|  | CYP2C18 | -1.58 | 0.022 | Cytochrome P450, family 2, subfamily C, polypeptide 18 |
|  | PPBP | 5.93 | 0.000 | Pro-platelet basic protein (chemokine (C-X-C motif) ligand 7) |
|  | PYY | -5.12 | 0.000 | Peptide tyrosine tyrosine |
|  |  |  |  |  |
| Overweight vs. Normal^†^ | | |  |  |
| CMS1 | TH | -3.00 | 0.003 | tyrosine hydroxylase |
|  | RBFOX1 | -5.47 | 0.000 | RNA binding fox-1 homolog 1 |
|  | SYN1 | -2.08 | 0.003 | synapsin I |
|  | UCHL1 | -2.11 | 0.010 | ubiquitin C-terminal hydrolase L1 |
|  | CALCA | -4.61 | 0.001 | calcitonin related polypeptide alpha |
|  | SCN2A | -2.58 | 0.033 | sodium voltage-gated channel alpha subunit 2 |
|  | NCAM1 | 2.17 | 0.009 | neural cell adhesion molecule 1 |
|  | SYP | 2.08 | 0.001 | synaptophysin |
|  | RIMBP2 | 2.82 | 0.004 | RIMS binding protein 2 |
|  | SNAP25 | 2.75 | 0.004 | synaptosome associated protein 25 |
|  |  |  |  |  |
| CMS2 | FBXO2 | 1.85 | 0.031 | F-box protein 2 |
|  | DRD4 | -1.00 | 0.049 | dopamine receptor D4 |
|  | TRIM71 | -3.45 | 0.014 | tripartite motif containing 71 |
|  | CXCL11 | 1.80 | 0.020 | C-X-C motif chemokine ligand 11 |
|  | CXCL9 | 2.06 | 0.003 | C-X-C motif chemokine ligand 9 |
|  | FBXW5 | -0.81 | 0.015 | F-box and WD repeat domain containing 5 |
|  | PPBP | 4.53 | 0.000 | pro-platelet basic protein |
|  | SPSB4 | -1.51 | 0.035 | splA/ryanodine receptor domain and SOCS box containing 4 |
|  | BDKRB1 | 1.18 | 0.015 | bradykinin receptor B1 |
|  | ASB12 | -1.45 | 0.041 | ankyrin repeat and SOCS box containing 12 |
|  |  |  |  |  |
| CMS3 | HP | -5.35 | 0.034 | haptoglobin |
|  | VTN | -3.74 | 0.022 | vitronectin |
|  | BPIFB1 | 16.20 | 0.000 | BPI fold containing family B member 1 |
|  | FGA | -8.19 | 0.000 | fibrinogen alpha chain |
|  | FGB | -7.64 | 0.003 | fibrinogen beta chain |
|  | PAX7 | -11.17 | 0.023 | paired box 7 |
|  | MAGEA11 | 20.54 | 0.000 | MAGE family member A11 |
|  | MAGEA12 | 20.33 | 0.000 | MAGE family member A12 |
|  | MAGEA3 | 23.84 | 0.000 | MAGE family member A3 |
|  | MAGEA6 | 23.68 | 0.000 | MAGE family member A6 |
|  |  |  |  |  |
| CMS4 | ANPEP | -2.07 | 0.025 | alanyl aminopeptidase, membrane |
|  | ALPI | -3.21 | 0.000 | alkaline phosphatase, intestinal |
|  | TDRD9 | 1.55 | 0.012 | tudor domain containing 9 |
|  | ABCG5 | -3.33 | 0.000 | ATP binding cassette subfamily G member 5 |
|  | TEX101 | 2.73 | 0.005 | testis expressed 101 |
|  | DSG1 | 2.87 | 0.000 | desmoglein 1 |
|  | SYCP2 | -1.85 | 0.009 | synaptonemal complex protein 2 |
|  | TDRD1 | 2.34 | 0.000 | tudor domain containing 1 |
|  | ABCG8 | -2.61 | 0.007 | ATP binding cassette subfamily G member 8 |
|  | MAEL | 2.30 | 0.047 | maelstrom spermatogenic transposon silencer |
|  |  |  |  |  |
| Obese vs. Overweight^†^ | | |  |  |
| CMS1 | APOH | -3.27 | 0.009 | apolipoprotein H |
|  | SAA1 | 4.11 | 0.002 | serum amyloid A1 |
|  | HPR | -6.39 | 0.003 | haptoglobin-related protein |
|  | IL10 | 2.82 | 0.000 | interleukin 10 |
|  | CCR2 | 2.29 | 0.025 | C-C motif chemokine receptor 2 |
|  | NCAM1 | -2.36 | 0.017 | neural cell adhesion molecule 1 |
|  | CCL13 | 2.71 | 0.033 | C-C motif chemokine ligand 13 |
|  | SNAP25 | -2.88 | 0.013 | synaptosome associated protein 25 |
|  | PEX3 | 0.85 | 0.019 | peroxisomal biogenesis factor 3 |
|  | CD274 | 2.22 | 0.004 | CD274 molecule |
|  |  |  |  |  |
| CMS2 | CALB1 | -4.22 | 0.032 | calbindin 1 |
|  | SOX2 | 4.27 | 0.017 | SRY-box transcription factor 2 |
|  | FSD1 | -1.59 | 0.021 | fibronectin type III and SPRY domain containing 1 |
|  | CLSTN2 | 2.86 | 0.001 | calsyntenin 2 |
|  | SLC26A4 | -2.43 | 0.001 | solute carrier family 26 member 4 |
|  | NKX2-1 | -8.82 | 0.000 | NK2 homeobox 1 |
|  | KCNQ2 | 2.21 | 0.020 | kinase non-catalytic C-lobe domain containing 1 |
|  | CTNNA2 | 3.84 | 0.006 | catenin alpha 2 |
|  | TBX3 | 2.07 | 0.000 | T-box transcription factor 3 |
|  | MMP8 | 7.18 | 0.000 | matrix metallopeptidase 8 |
|  |  |  |  |  |
| CMS3 | GPR37L1 | -1.41 | 0.010 | G protein-coupled receptor 37 like 1 |
|  | PYY | -6.71 | 0.000 | peptide YY |
|  | INSL5 | -5.47 | 0.008 | insulin like 5 |
|  | SST | -3.85 | 0.017 | somatostatin |
|  | SSTR2 | -2.58 | 0.000 | somatostatin receptor 2 |
|  | SSTR5 | 3.67 | 0.005 | somatostatin receptor 5 |
|  | CCR2 | -2.09 | 0.011 | C-C motif chemokine receptor 2 |
|  | CCL13 | -2.50 | 0.013 | C-C motif chemokine ligand 13 |
|  | NPW | 2.19 | 0.049 | neuropeptide W |
|  | CCR10 | -1.62 | 0.022 | C-C motif chemokine receptor 10 |
|  |  |  |  |  |
| CMS4 | PYY | -4.25 | 0.000 | peptide YY |
|  | GPR18 | -2.19 | 0.000 | G protein-coupled receptor 18 |
|  | INSL5 | -3.40 | 0.020 | insulin like 5 |
|  | NPW | 1.72 | 0.010 | neuropeptide W |
|  | PPBP | 2.32 | 0.006 | pro-platelet basic protein |
|  | NMUR2 | 3.55 | 0.004 | neuromedin U receptor 2 |
|  | TAS2R4 | -1.37 | 0.007 | taste 2 receptor member 4 |
|  | TAS2R14 | -1.16 | 0.003 | taste 2 receptor member 14 |
|  | TAS2R30 | -4.47 | 0.001 | taste 2 receptor member 30 |
|  | TAS2R31 | -2.89 | 0.000 | taste 2 receptor member 31 |

* Log2 fold change (Log2FC) from the DESeq2-obtained DEGs

**^∇^** False discovery rate P value (Padj) from the DESeq2-obtained DEGs

^†^ BMI 19-24.9 (Normal), BMI 25-29.9 (Overweight), BMI ≥30 (Obese)
