# Supplementary material for: Consensus molecular subtype differences linking colon adenocarcinoma and obesity revealed by a cohort transcriptomic analysis
Source: PLoS One. 2022 May 13;17(5):e0268436. doi: 10.1371/journal.pone.0268436 (PMC9106217; doi:10.1371/journal.pone.0268436)
Supplement: S3 Table — (DOCX) [file pone.0268436.s003.docx]

Supplemental Table 3. Sensitivity analysis to assess maximal clique centrality (MCC) identified hub genes in four additional topographical algorithms.

|  | **Obese vs Normal** | | | | | **Obese vs Overweight** | | | | | **Overweight vs Normal** | | | | |
| --- | --- | --- | --- | --- | --- | --- | --- | --- | --- | --- | --- | --- | --- | --- | --- |
|  | **MCC*** | **DMNC** | **MNC** | **Degree** | **EPC** | **MCC** | **DMNC** | **MNC** | **Degree** | **EPC** | **MCC** | **DMNC** | **MNC** | **Degree** | **EPC** |
| **CMS1** | HSD17B1 | KIF1A | SYP | SYN2 | SYN2 | SNAP25 | JAK3 | RAB3C | SCN2A | SCN2A | SCN2A | TH | RBFOX1 | RBFOX1 | RBFOX1 |
|  | SYP^†^ | INHA | CPLX2 | CPLX2 | NRXN3 | NCAM1 | CADPS | SCN2A | CADPS | GABRB2 | RIMBP2 | UNC13A | TH | TH | TH |
|  | KIF1A | PIK3R1 | SYN2 | PIK3R1 | CPLX2 | CD274 | EMR1 | SCNN1G | GABRB2 | CD274 | RBFOX1 | SCN2A | SNAP25 | SNAP25 | SNAP25 |
|  | CPLX2 | ALPPL2 | CYP1A1 | KIF1A | SYP | IL10 | NPY1R | GABRB2 | CD274 | CALB2 | SNAP25 | SPRR2A | RIMBP2 | RIMBP2 | RIMBP2 |
|  | SYN2 | OXTR | RAB3C | SYP | KIF1A | CCR2 | PEX3 | CD274 | SNAP25 | SNAP25 | UCHL1 | DSC3 | SYP | SYP | SYP |
|  | CYP1A1 | RAB3C | SYNPR | IL10 | RBFOX1 | CCL13 | CCL13 | SNAP25 | NCAM1 | CCL13 | SYN1 | CALCA | NCAM1 | NCAM1 | NCAM1 |
|  | RAB3C^‡^ | BSN | UNC13A | RBFOX1 | BSN | SAA1 | SAA2 | NCAM1 | KIF1A | NCAM1 | TH | KRT4 | SCN2A | CALCA | SCN2A |
|  | SYNPR | UNC13A | BSN | SIRT1 | UNC13A | PEX3 | HPR | CCR2 | CCR2 | CCR2 | CALCA | NEUROD1 | CALCA | UCHL1 | CALCA |
|  | UNC13A | CYP17A1 | UGT1A6 | CALCA | SYNPR | HPR | SAA1 | SCNN1B | IL10 | IL10 | NCAM1 | C5 | UCHL1 | NEUROD1 | UCHL1 |
|  | BSN | PSCA | RBFOX1 | RAB3C | RAB3C | APOH | NPW | IL10 | SAA1 | SAA1 | SYP | SPRR3 | SYN1 | SYN1 | SYN1 |
|  |  |  |  |  |  |  |  |  |  |  |  |  |  |  |  |
| **CMS2** | APOA2 | APOA2 | APOA2 | APOA2 | KIT | CALB1 | CALB1 | CALB1 | CALB1 | CALB1 | BDKRB1 | BDKRB1 | BDKRB1 | BDKRB1 | CXCL11 |
|  | DNMT3A | MKRN3 | NPHS1 | DNMT3A | APOA2 | SOX2 | C5orf58 | SOX2 | SOX2 | PCDH19 | PPBP | PPBP | PPBP | PPBP | BDKRB1 |
|  | TWIST1 | NPHS1 | CTCFL | TWIST1 | DNMT3A | FSD1 | SOX2 | FSD1 | FSD1 | SOX2 | FBXW5 | FBXW5 | FBXW5 | SPSB4 | DNMT3A |
|  | CYP1A1 | NLRP6 | CYP1A1 | CYP1A1 | CYP17A1 | CLSTN2 | FSD1 | CLSTN2 | CLSTN2 | CLSTN2 | CXCL9 | CXCL9 | CXCL9 | TRIM71 | PPBP |
|  | ALDH1A2 | CTCFL | TWIST1 | ALDH1A2 | CYP1A1 | SLC26A4 | CLSTN2 | ATP12A | SLC26A4 | SLC26A4 | SPSB4 | SPSB4 | SPSB4 | FBXW11 | CXCL9 |
|  | WNT6 | CYP1A1 | ALDH1A2 | WNT6 | ALDH1A2 | NKX2-1 | SLC26A4 | SLC26A4 | NKX2-1 | NKX2-1 | TRIM71 | TRIM71 | TRIM71 | IGF2 | TGFB2 |
|  | UGT2B10 | ALDH1A2 | WNT6 | UGT2B10 | UGT2B10 | KCNQ2 | NKX2-1 | NKX2-1 | KCNQ2 | KCNQ2 | FBXW11 | FBXW11 | FBXW11 | FBXO2 | IGF2 |
|  | GPC3 | UGT2B10 | UGT2B10 | GPC3 | GPC3 | CTNNA2 | TBX3 | GLIPR1 | CTNNA2 | CTNNA2 | FBXO2 | FBXO2 | FBXO2 | TAS2R31 | DRD4 |
|  | ZIC2 | GPC3 | GPC3 | ZIC2 | GPR83 | TBX3 | PLA2G2A | TBX3 | TBX3 | KIF5C | TAS2R31 | TAS2R31 | TAS2R31 | SOX2 | TAS2R31 |
|  | NTS | LAMB1 | LAMB1 | NTS | LAMB1 | MMP8 | FXYD2 | FXYD2 | MMP8 | TBX3 | ASB12 | ASB12 | ASB12 | ASB12 | SOX2 |
|  |  |  |  |  |  |  |  |  |  |  |  |  |  |  |  |
| **CMS3** | KRT24 | KRT24 | KRT24 | KRT24 | KRT24 | GPR37L1 | GPR37L1 | NRXN1 | NRXN1 | IGF2 | HP | HP | HP | HP | HP |
|  | MAGEA6 | MAGEA6 | MAGEA6 | MAGEA6 | MAGEA6 | PYY | PYY | FGB | IGF2 | PYY | MAGEA3 | MAGEA3 | MAGEA3 | MAGEA3 | MAGEA3 |
|  | IRX2 | MAGEA3 | IRX2 | IRX2 | IRX2 | INSL5 | INSL5 | PYY | DRD5 | SST | MAGEA6 | CYP2B6 | MAGEA6 | MAGEA6 | MAGEA6 |
|  | MAGEA3 | MAGEA12 | MAGEA3 | MAGEA3 | MAGEA3 | SST | CCL13 | GCG | SST | GCG | MAGEA11 | MAGEA6 | MAGEA11 | MAGEA11 | MAGEA11 |
|  | MAGEA12 | TENM2 | MAGEA12 | MAGEA12 | MAGEA12 | CCR2 | SSTR5 | SST | GCG | TIMP1 | PAX7 | BEST4 | PAX7 | PAX7 | PAX7 |
|  | TENM2 | MT4 | TENM2 | TENM2 | TENM2 | CCL13 | NPW | FGA | FGA | SSTR5 | FGB | KRT24 | FGB | FGB | FGB |
|  | MT4 | TAS2R30 | MT4 | MT4 | MT4 | SSTR5 | CCR10 | CCR2 | TIMP1 | CCR2 | FGA | FGB | FGA | FGA | FGA |
|  | TAS2R30 | CTCFL | TAS2R30 | TAS2R30 | TAS2R30 | NPW | MMRN1 | SSTR5 | SSTR5 | CCL13 | BPIFB1 | FGA | BPIFB1 | BPIFB1 | BPIFB1 |
|  | HOXC13 | HOXC13 | HOXC13 | HOXC13 | HOXC13 | CCR10 | SSTR2 | SSTR2 | CCR2 | CCR10 | VTN | VTN | VTN | VTN | VTN |
|  | MAGEA11 | OLFM1 | MAGEA11 | MAGEA11 | MAGEA11 | SSTR2 | FIGF | CYP3A4 | SSTR2 | SSTR2 | MAGEA12 | MAGEA12 | MAGEA12 | MAGEA12 | MAGEA12 |
|  |  |  |  |  |  |  |  |  |  |  |  |  |  |  |  |
| **CMS4** | UGT1A10 | ABCG2 | NR1H4 | NR1H4 | ABCG2 | PYY | GPR18 | PYY | PYY | PYY | TDRD1 | TEX101 | TDRD1 | DSG1 | DSG1 |
|  | NR1H4 | GRM4 | CCL25 | CHGA | NR1H4 | INSL5 | TAS2R20 | INSL5 | INSL5 | INSL5 | SYCP2 | TDRD9 | SYCP2 | TEX101 | ALPI |
|  | PYY | NPW | GCG | GCG | GCG | GPR18 | PPBP | TAS2R20 | TAS2R20 | GPR18 | TEX101 | ALPI | TEX101 | ALPI | ANPEP |
|  | CYP2C18 | UGT1A10 | CYP2C18 | CYP2C9 | CYP2C18 | TAS2R20 | TAS2R31 | PPBP | PPBP | TAS2R20 | DSG1 | ANPEP | TDRD9 | ANPEP | SLC22A4 |
|  | CYP2C9 | ABCG8 | CYP2C9 | GPT | CYP2C9 | PPBP | TAS2R14 | TAS2R31 | TAS2R31 | PPBP | TDRD9 | TREH | ABCG5 | MAEL | SLC15A1 |
|  | UGT1A6 | TAS2R30 | UGT1A6 | UGT1A6 | GPT | TAS2R31 | TAS2R13 | TAS2R13 | TAS2R13 | TAS2R31 | ALPI | MTTP | SBSN | SLC15A1 | ABCG2 |
|  | PPBP | PYY | PPBP | UGT1A1 | UGT1A6 | TAS2R13 | TAS2R4 | TAS2R4 | NMUR2 | TAS2R4 | ABCG5 | CETP | TREH | ABCG2 | SI |
|  | UGT1A1 | FMO1 | UGT1A1 | UGT1A8 | UGT1A1 | TAS2R4 | TAS2R30 | NMUR2 | CHGA | NMUR2 | MAEL | MAEL | TGM3 | SI | KRT17 |
|  | UGT1A8 | INSL5 | UGT1A8 | EGF | UGT1A8 | NMUR2 | NPW | TAS2R19 | TAS2R19 | NPW | SI | SI | MAEL | EGF | EGF |
|  | CYP1A1 | SULT1A2 | CYP1A1 | CYP1A1 | CYP1A1 | TAS2R19 | TAS2R19 | GCG | GCG | GCG | ABCG8 | ABCG8 | ABCG8 | ABCG8 | ABCG8 |

*Topological algorithms: maximal clique centrality (MCC), Degree, Edge Percolated Component (EPC), Maximum Neighborhood Component (MNC), and Density of Maximum Neighborhood Component (DMNC)

^†^Blue font indicates common in four of the five algorithms

^‡^Red font indicates common in all five algorithms
