# Supplementary material for: Consensus molecular subtype differences linking colon adenocarcinoma and obesity revealed by a cohort transcriptomic analysis
Source: PLoS One. 2022 May 13;17(5):e0268436. doi: 10.1371/journal.pone.0268436 (PMC9106217; doi:10.1371/journal.pone.0268436)
Supplement: S4 Table — (DOCX) [file pone.0268436.s004.docx]

Supplementary Table 4. Predicted drug sensitivity for normal compared to obese BMI categories.

| **Name** | **Targets** | **Target pathway** | **log(IC50) Normal - log(IC50) Obese** | **p-value** | **CMS** |
| --- | --- | --- | --- | --- | --- |
| GNF-2 | BCR-ABL | ABL signaling | 0.058 | 0.026 | CMS4 |
| Obatoclax Mesylate | BCL2, BCL-XL, BCL-W, MCL1 | Apoptosis regulation | 0.698 | 0.013 | CMS1 |
| Palbociclib | CDK4, CDK6 | Cell cycle | 0.356 | 0.017 | CMS1 |
| CGP-082996 | CDK4 | Cell cycle | 0.176 | 0.021 | CMS2 |
| RO-3306 | CDK1 | Cell cycle | 0.132 | 0.036 | CMS4 |
| Parthenolide | HDAC1 | Chromatin histone acetylation | 0.118 | 0.039 | CMS4 |
| IPA-3 | PAK1 | Cytoskeleton | 0.331 | 0.023 | CMS1 |
| PF-562271 | FAK, FAK2 | Cytoskeleton | 0.244 | 0.022 | CMS1 |
| Gemcitabine | Pyrimidine antimetabolite | DNA replication | 1.232 | 0.028 | CMS1 |
| Bleomycin | dsDNA break induction | DNA replication | 1.092 | 0.024 | CMS1 |
| Doxorubicin | Anthracycline | DNA replication | 0.575 | 0.010 | CMS1 |
| Methotrexate | Antimetabolite | DNA replication | 0.281 | 0.030 | CMS2 |
| Lapatinib | EGFR, ERBB2 | EGFR signaling | 0.161 | 0.024 | CMS4 |
| PLX-4720 | BRAF | ERK MAPK signaling | 0.311 | 0.041 | CMS1 |
| KU-55933 | ATM | Genome integrity | -0.032 | 0.008 | CMS1 |
| JNK Inhibitor VIII | JNK | JNK and p38 signaling | 0.053 | 0.031 | CMS4 |
| DMOG | HIF-PH | Metabolism | 0.503 | 0.027 | CMS1 |
| FH535 | PPARgamma, PPARdelta | Metabolism | -0.168 | 0.034 | CMS2 |
| FH535 | PPARgamma, PPARdelta | Metabolism | -0.123 | 0.014 | CMS4 |
| ZM447439 | AURKA, AURKB | Mitosis | 0.324 | 0.034 | CMS1 |
| Vinorelbine | Microtubule destabiliser | Mitosis | 0.086 | 0.039 | CMS1 |
| Thapsigargin | SERCA | Other | 0.909 | 0.031 | CMS1 |
| Tipifarnib | Farnesyl-transferase (FNTA) | Other | 0.427 | 0.027 | CMS1 |
| Midostaurin | PKC, PPK, FLT1, c-FGR, others | Other | 0.396 | 0.021 | CMS1 |
| Shikonin | not defined | Other | 0.050 | 0.014 | CMS1 |
| Bexarotene | Retinioic X receptor (RXR) agonist | Other | -0.156 | 0.011 | CMS3 |
| Ponatinib | ABL, PDGFRA, VEGFR2, FGFR1, SRC, TIE2, FLT3 | Other, kinases | 0.355 | 0.049 | CMS1 |
| Dasatinib | ABL, SRC, Ephrins, PDGFR, KIT | Other, kinases | 0.463 | 0.046 | CMS4 |
| BMS-509744 | ITK | Other, kinases | 0.146 | 0.015 | CMS4 |
| Bosutinib | SRC, ABL, TEC | Other, kinases | 0.057 | 0.040 | CMS4 |
| Serdemetan | MDM2 | p53 pathway | 0.349 | 0.014 | CMS1 |
| Temsirolimus | MTOR | PI3K/MTOR signaling | 0.519 | 0.003 | CMS1 |
| AZD8055 | MTORC1, MTORC2 | PI3K/MTOR signaling | 0.430 | 0.000 | CMS1 |
| Dactolisib | PI3K (class 1), MTORC1, MTORC2 | PI3K/MTOR signaling | 0.390 | 0.042 | CMS1 |
| Temsirolimus | MTOR | PI3K/MTOR signaling | 0.427 | 0.013 | CMS4 |
| Elesclomol | HSP90 | Protein stability and degradation | 0.615 | 0.021 | CMS1 |
