# Supplementary material for: Consensus molecular subtype differences linking colon adenocarcinoma and obesity revealed by a cohort transcriptomic analysis
Source: PLoS One. 2022 May 13;17(5):e0268436. doi: 10.1371/journal.pone.0268436 (PMC9106217; doi:10.1371/journal.pone.0268436)
Supplement: S5 Table — (DOCX) [file pone.0268436.s005.docx]

Supplementary Table 5. Predicted drug sensitivity for normal compared to overweight BMI categories.

| **Name** | **Targets** | **Target pathway** | **log(IC50) Normal - log(IC50) Obese** | **p-value** | **CMS** |
| --- | --- | --- | --- | --- | --- |
| RO-3306 | CDK1 | Cell cycle | 0.2360 | 0.002 | CMS2 |
| CGP-082996 | CDK4 | Cell cycle | 0.1739 | 0.020 | CMS2 |
| Camptothecin | TOP1 | DNA replication | 0.5147 | 0.041 | CMS2 |
| Methotrexate | Antimetabolite | DNA replication | 0.2221 | 0.017 | CMS2 |
| Afatinib | ERBB2, EGFR | EGFR signaling | 0.2826 | 0.012 | CMS2 |
| Gefitinib | EGFR | EGFR signaling | 0.0796 | 0.000 | CMS2 |
| KU-55933 | ATM | Genome integrity | 0.2150 | 0.001 | CMS2 |
| Bicalutamide | AR | Hormone-related | -0.0598 | 0.001 | CMS2 |
| JNK Inhibitor VIII | JNK | JNK and p38 signaling | 0.0788 | 0.004 | CMS2 |
| Doramapimod | p38, JNK2 | JNK and p38 signaling | 0.0640 | 0.014 | CMS2 |
| AS601245 | JNK1, JNK2, JNK2 | JNK and p38 signaling | -0.4234 | 0.023 | CMS3 |
| FH535 | PPARgamma, PPARdelta | Metabolism | -0.2210 | 0.009 | CMS2 |
| Tretinoin | Retinoic acid | Other | 0.1998 | 0.004 | CMS2 |
| FTI-277 | Farnesyl-transferase (FNTA) | Other | -0.0904 | 0.039 | CMS2 |
| Bosutinib | SRC, ABL, TEC | Other, kinases | -0.3230 | 0.029 | CMS1 |
| SL0101 | RSK, AURKB, PIM1, PIM3 | Other, kinases | 0.1593 | 0.031 | CMS2 |
| Bosutinib | SRC, ABL, TEC | Other, kinases | 0.0804 | 0.008 | CMS2 |
| Sunitinib | PDGFR, KIT, VEGFR, FLT3, RET, CSF1R | RTK signaling | 0.0503 | 0.031 | CMS2 |
| GW441756 | NTRK1 | RTK signaling | 0.0752 | 0.020 | CMS3 |
| NVP-TAE684 | ALK | RTK signaling | -0.0744 | 0.037 | CMS3 |
| CHIR-99021 | GSK3A, GSK3B | WNT signaling | -0.1717 | 0.032 | CMS2 |
