# Supplementary material for: Consensus molecular subtype differences linking colon adenocarcinoma and obesity revealed by a cohort transcriptomic analysis
Source: PLoS One. 2022 May 13;17(5):e0268436. doi: 10.1371/journal.pone.0268436 (PMC9106217; doi:10.1371/journal.pone.0268436)

Supplemental Figure 1

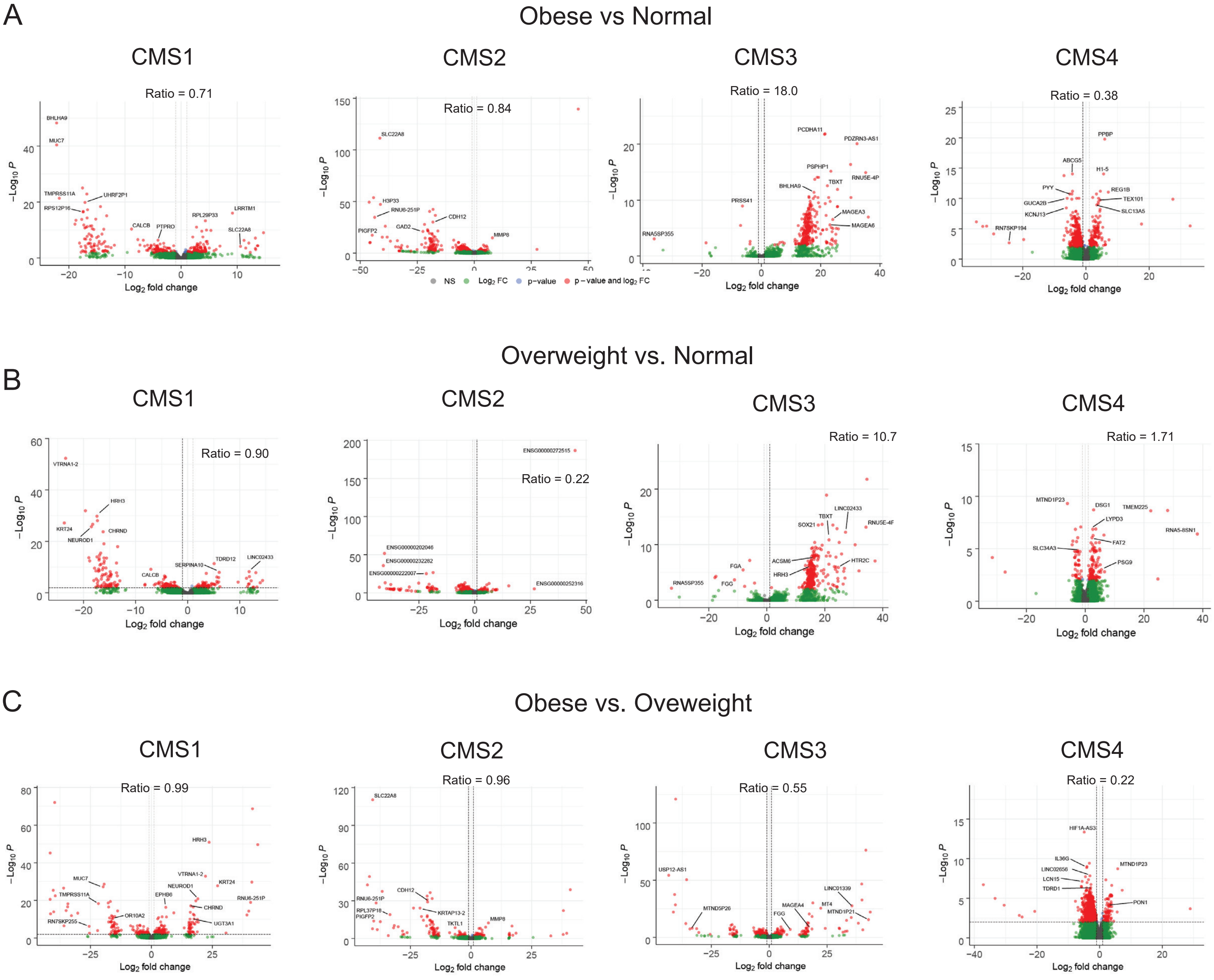

Supplement: S1 Fig — (A) Volcano plots were used to visualize DESeq2-obtained DEGs (MeanBase > 10, FDR p value < 0.05) between Obese vs. Normal (A), Overweight vs Normal (B), and Obese vs. Overweight (C) comparisons for each CMS category. The R package EnhancedVolcano was used to construct the plots. The ratio of overexpressed to underexpressed DEGs is shown for each volcano plot. The DEGs with a false discovery rate less than 0.05 are shown as red dots while nonsignificant DEGs are represented as green dots. Select highly significant and differentially expressed genes are identified in the plots. (PDF) [file pone.0268436.s006.pdf]
