# Supplementary material for: Consensus molecular subtype differences linking colon adenocarcinoma and obesity revealed by a cohort transcriptomic analysis
Source: PLoS One. 2022 May 13;17(5):e0268436. doi: 10.1371/journal.pone.0268436 (PMC9106217; doi:10.1371/journal.pone.0268436)

Supplemental Figure 2

Obesity vs Normal DEGs Across CMS Catergories

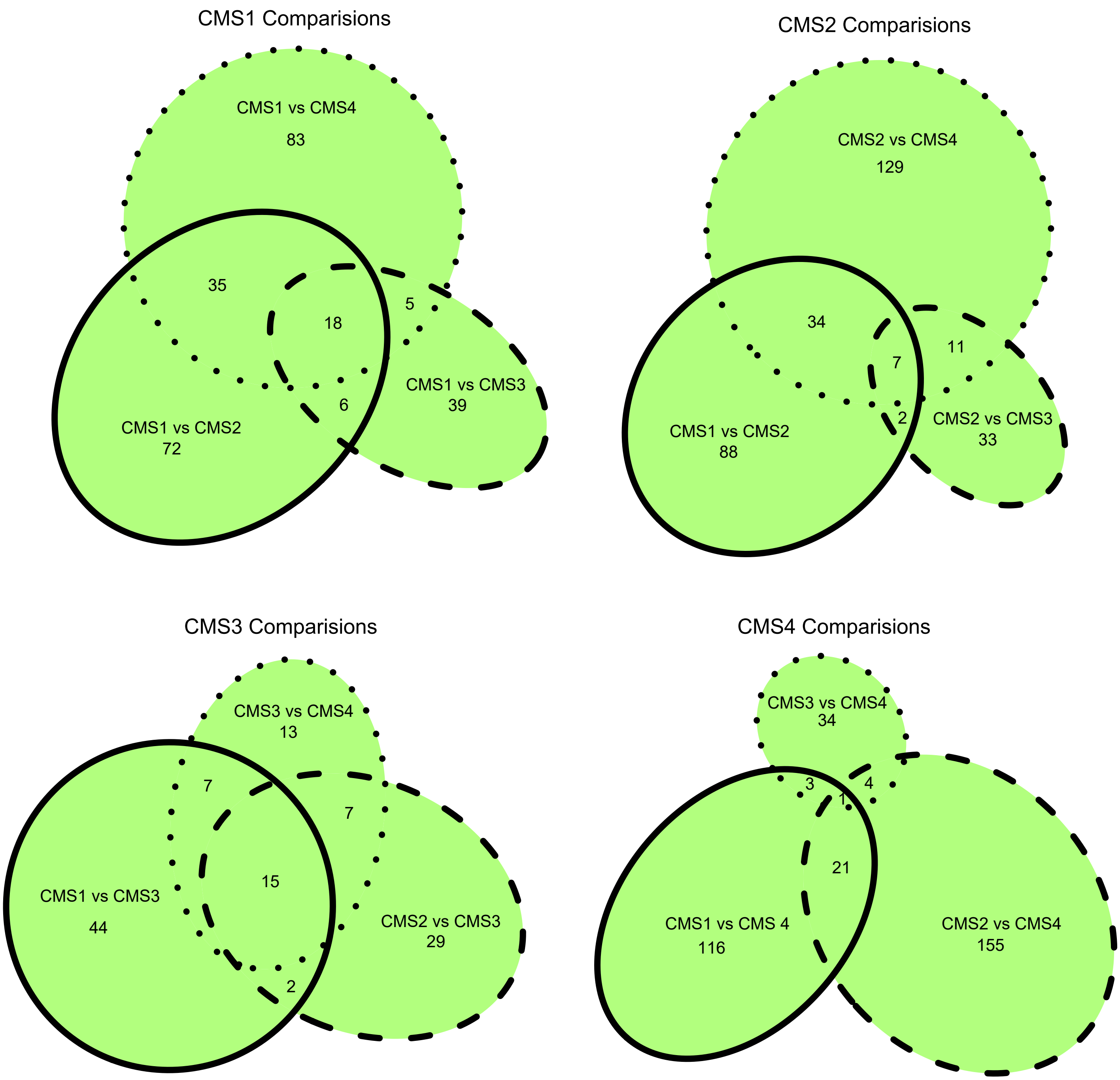

Supplement: S2 Fig — Euler diagrams were used to visualize the weighted overlap of DESeq2-obtained Obese vs. Normal DEGs (MeanBase > 10, FDR p value < 0.05) using an interaction term for obesity:CMS in the DESeq2 linear model for each CMS category. The R package eulerr was used to construct the Euler diagrams. (PDF) [file pone.0268436.s007.pdf]
