# Supplementary figures and images for: Consensus molecular subtype differences linking colon adenocarcinoma and obesity revealed by a cohort transcriptomic analysis
Source: PLoS One. 2022 May 13;17(5):e0268436. doi: 10.1371/journal.pone.0268436 (PMC9106217; doi:10.1371/journal.pone.0268436)

Supplemental Figure 3

A Overall Survival

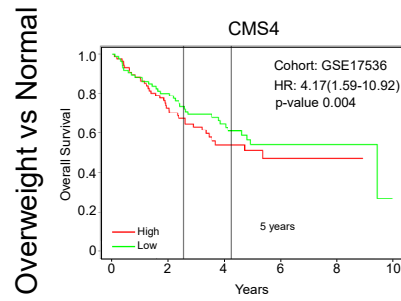

B Relapse-free Survival

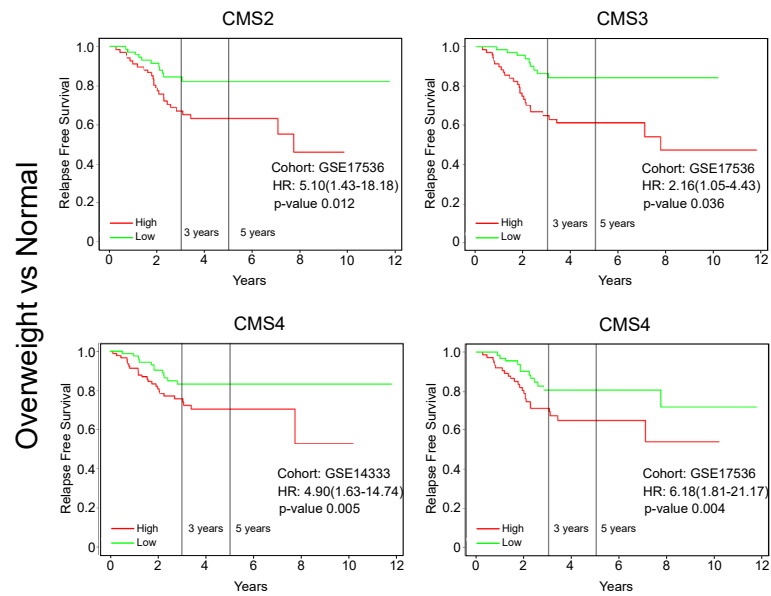

Supplement: S3 Fig — The expression of the top 20 significantly upregulated DESeq2-obtained DEGs (MeanBase > 10, FDR p value < 0.05) in the overweight vs. normal comparisons for each CMS category were assessed in GSE17536 and GSE41258 for overall survival (A) and GSE14333 and GSE17536 for relapse-free survival (B) using the PROGgeneV2 tool. Survival analyses were adjusted for age, stage, and gender covariates and bifurcated based on median expression. The hazard ratios, 95% confidence intervals, and p values were reported for the Kaplan-Meier plots. (PDF) [file pone.0268436.s008.pdf]

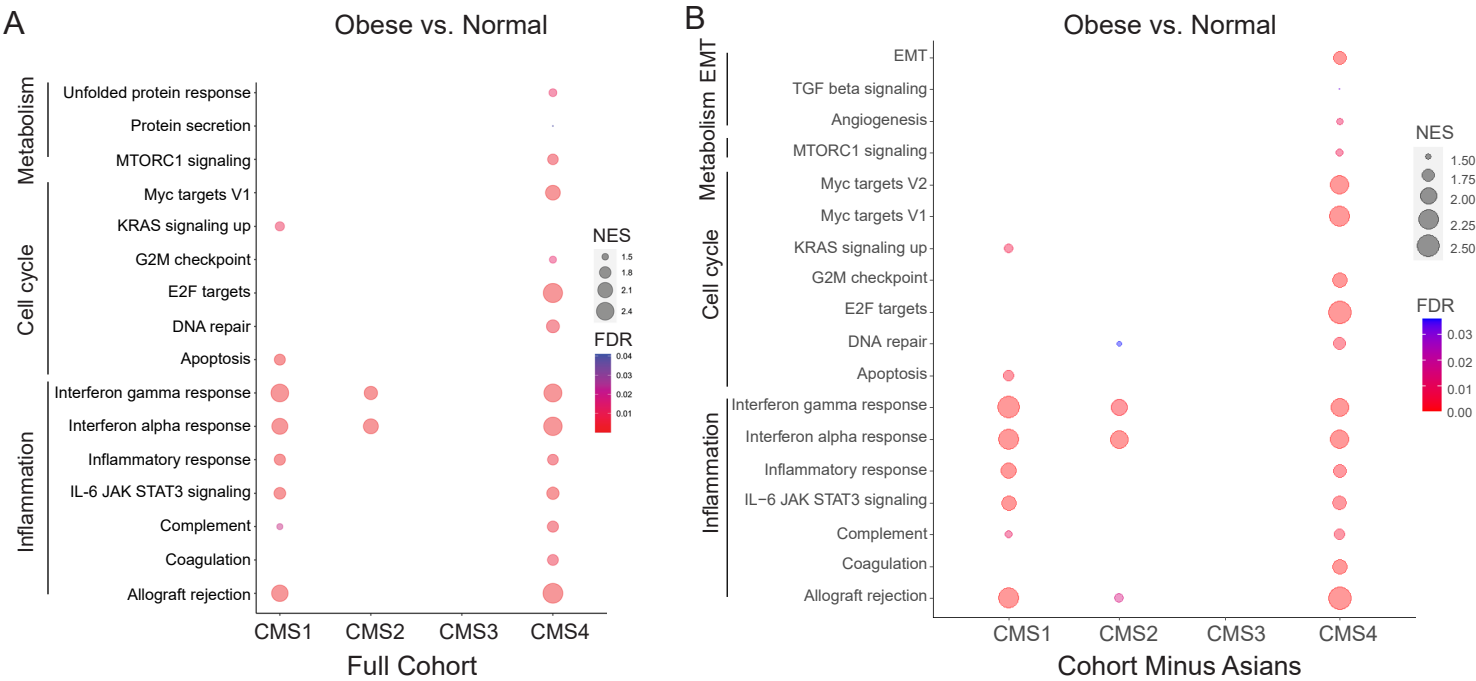

Supplement: S4 Fig — Normalized RNA-seq counts obtained from DESeq2 were used for Gene Set Enrichment Analysis (GSEA). Hallmark gene sets were assessed in the obese vs. normal comparisons in the whole population (A) and the population without the Asian patients (B) for each CMS category. Bubbles for gene sets with a false discovery rate q-value less than 0.05 were reported. The size of bubbles represents the normalized enrichment score (NES). The color of bubbles represents false discovery rate q-value (FDR). (PDF) [file pone.0268436.s009.pdf]

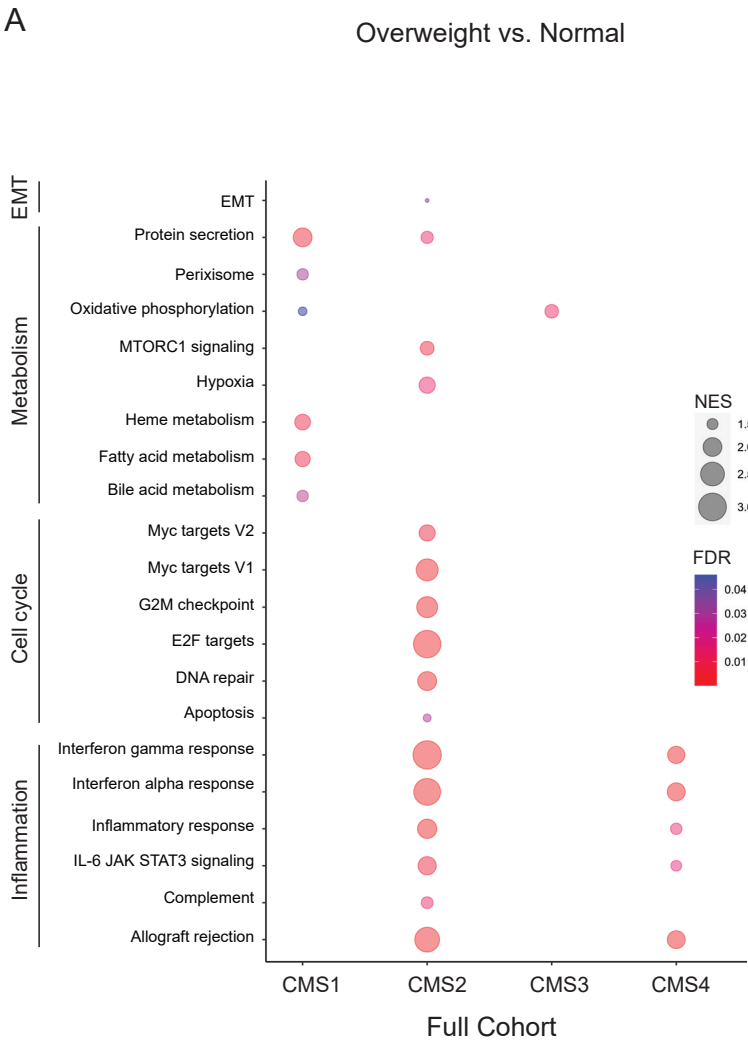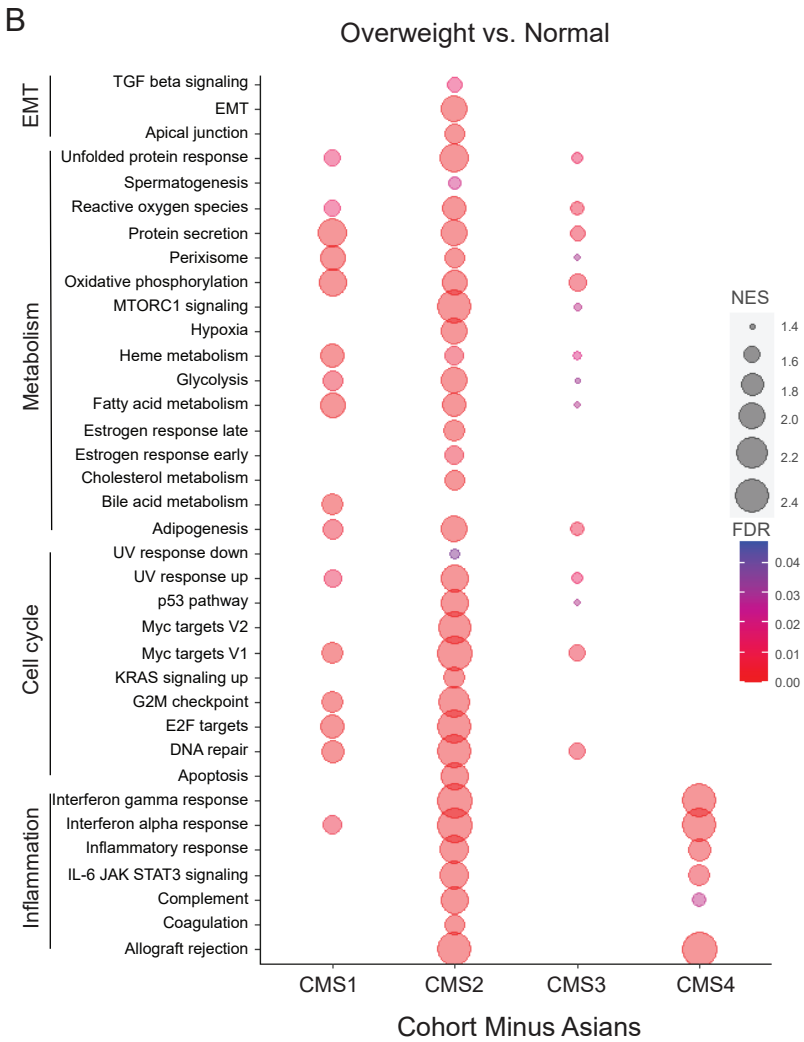

Supplement: S5 Fig — Normalized RNA-seq counts obtained from DESeq2 were used for Gene Set Enrichment Analysis (GSEA). Hallmark gene sets were assessed in the overweight vs. normal comparison in the whole population (A) and the population without the Asian patients (B) for each CMS category. Bubbles for gene sets with a false discovery rate q-value less than 0.05 were reported. The size of bubbles represents the normalized enrichment score (NES). The color of bubbles represents false discovery rate q- value (FDR). (PDF) [file pone.0268436.s010.pdf]

Obese Hub genes across BMI categories

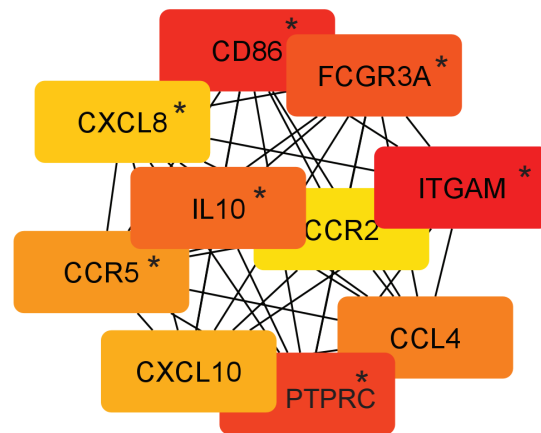

Supplement: S7 Fig — Obesity-linked DEGs (MeanBase > 10, FDR p value < 0.05) obtained using the Likelihood ratio test in DESeq2 were used to construct a protein-protein interaction (PPI) network from the STRING database in Cytoscape. The Cytohubba package in Cytoscape was used to perform the hub gene analysis from the PPI network for the obese vs. normal comparison. Hub genes were identified using the maximal clique centrality (MCC) topological algorithm to obtain the top 10 ranked genes in all modules. Hub genes identified in at least three of the four topological algorithms are designated with an asterisk. The intensity and color (high, red; orange, medium, yellow, low) of the hub genes is shown. (PDF) [file pone.0268436.s012.pdf]

A

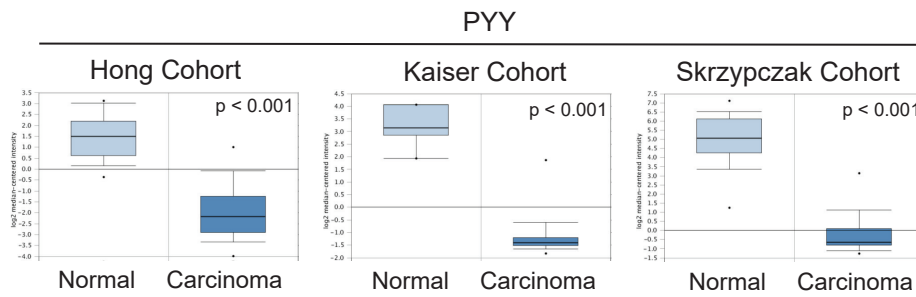

B

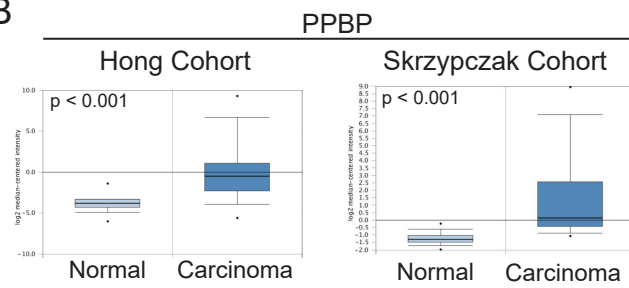

C

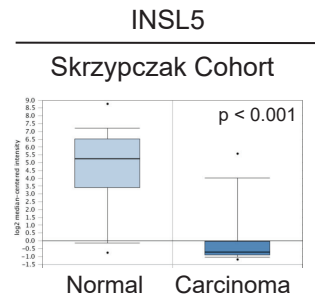

D

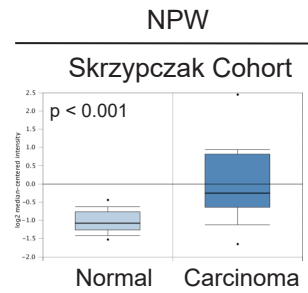

Supplement: S8 Fig — Hub genes were queried for mRNA expression using the Oncomine database. Hub gene expression in colon adenocarcinoma (Carcinoma) versus normal patient samples from the Hong Colorectal (Normal, n = 12; Carcinoma, n = 70), Skrzypczak Colorectal (Normal, n = 24; Carcinoma, n = 36), and Kaiser Colorectal (Normal, n = 5; Carcinoma, n = 41) cohorts. The p value from t-tests are shown. (A) PYY, (B) PPBP, (C) INSL5, and (D) NPW. (PDF) [file pone.0268436.s013.pdf]
