# Supplementary material for: Consensus molecular subtype differences linking colon adenocarcinoma and obesity revealed by a cohort transcriptomic analysis
Source: PLoS One. 2022 May 13;17(5):e0268436. doi: 10.1371/journal.pone.0268436 (PMC9106217; doi:10.1371/journal.pone.0268436)

Supplemental Figure 6

CMS1 Overweight vs Normal

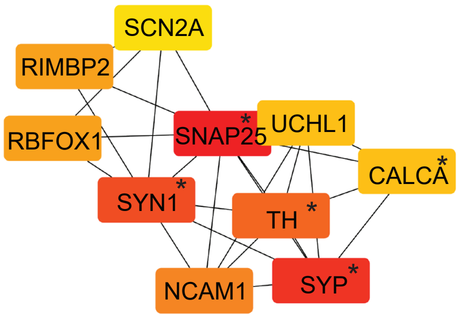

CMS2 Overweight vs Normal

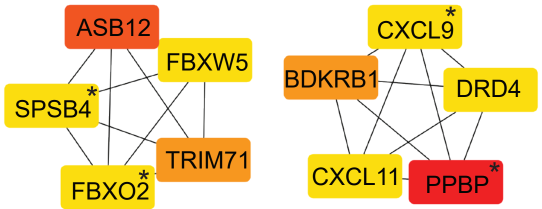

CMS3 Overweight vs Normal

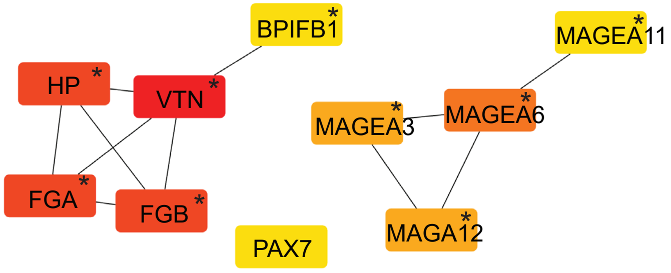

CMS4 Overweight vs Normal

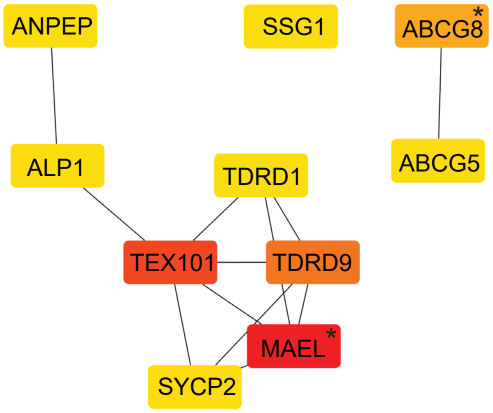

Supplement: S6 Fig — DESeq2-obtained DEGs (MeanBase > 10, FDR p value < 0.05) were used to construct a protein-protein interaction (PPI) network from the STRING database in Cytoscape. The Cytohubba package in Cytoscape was used to perform the hub gene analysis from the PPI network for the overweight vs. normal comparison for each CMS category. Hub genes were identified using the maximal clique centrality (MCC) topological algorithm to obtain the top 10 ranked genes in all modules. Hub genes identified in at least three of the four topological algorithms are designated with an asterisk. The intensity and color (high, red; orange, medium, yellow, low) of the hub genes is shown. (PDF) [file pone.0268436.s011.pdf]
