# Supplementary material for: Consensus molecular subtype differences linking colon adenocarcinoma and obesity revealed by a cohort transcriptomic analysis
Source: PLoS One. 2022 May 13;17(5):e0268436. doi: 10.1371/journal.pone.0268436 (PMC9106217; doi:10.1371/journal.pone.0268436)
Supplement: Attachment — Submitted filename: Greene_2021_PLOS_ONE_obesity and consensus molecular subtypes.docx [file pone.0268436.s014.docx]

The paper by Greene and colleagues reports interesting results of gene expression data by BMI categories, stratified by consensus-molecular subtypes (CMS) categories of colorectal cancer patients. A number of differentially expressed genes between obese/overweight and normal patients emerged, with some suggestions for differential associations with survival and drug sensitivity. The work is thorough and well conducted and explained. The manuscript is well written. My only concern is regarding the broad conclusions reached by investigating a relatively small sample set (especially within some CMS categories) and potential confounding, specifically by tumor stage, that needs to be addressed. Conclusions need to be less far-reaching.

Overall, a valuable contribution to the literature.

1) Abstract: please rephrase the conclusions to discuss “possible associations” rather than “effects”.
2) Patients: “Samples with an FDR greater than 0.05 were not classified”. Please add how many these were.
3) Considering the potential for confounding by race/ethnicity (e.g., Asian patients not represented among the obese), please perform a sensitivity analysis of the main findings, restricting the sample set to those from Caucasian patients.
4) Can results be adjusted for race/ethnicity?
5) Please add a table that illustrates what factors were associated with the CMS subtypes (similar to Table 1, but columns as CMS)
6) There could be confounding by tumor stage, especially because stage IV patients frequently present clinically after weight loss. Can the analysis of DEGs and GSEA be adjusted for tumor stage, to evaluate robustness? If not, can you exclude stage IV patients? Also, are CMS1 patients generally of lower age (and, accordingly, lower BMI) because they are more likely to have familial disease? If they are younger, please discuss this in the limitation section.
7) While intriguing, the results of DEGs may also be somewhat random. Please be more cautious in the interpretation.
8) Consider streamlining the text description of the GSEA results
9) It appears that most of the signals are appearing in CMS4 and less CMS3. This points toward greater impact of inflammation in the adipose tissue as a driver, rather than metabolic differences. The authors might want to consider making this point more clearly
10) Discussion: “These findings suggest a differential role of inflammation in the subtypes… whether the patient is overweight or obese” – from my read of the data it looks more like there was no substantial distinction between overweight and obese (e.g., in the direct comparison), but clearly a difference to normal. Perhaps other data are meant? Please clarify.
11) Paragraph “The deregulation of cellular energetics” should be worded more cautiously, because this was limited only to the CMS4 subtype.
12) Sentence “obesity may modulate the derivation of CMS3 and CMS4 tumors from canonical CMS2 tumors”. The evidence appears to be much stronger for CMS4. Please make that distinction clear. Overall, CMS3 does not emerge with strong signals from what I see?
13) Conclusion: Please rephrase first sentence to avoid causality (e.g., to “Our findings suggest that obesity is associated with CMS-specific CRC tumors”) and overall reduce the claims made in the conclusions, considering the limitations of the study.
14) Figure 3: Please make clear (including in the legend) why not all CMS are shown. These results are based on small numbers and should be interpreted with caution.

Minor comments:

1) Some additional references to add
   a. to refs 22-24 on adipose tissue and other mechanisms of energy balance and gastrointestinal cancer: Ulrich et al. *Nat Rev Gastroenterol Hepatol*. 2018;15:683-98.
   b. Also: Haffa et al: *J Clin Endocrinol Metab*. 2019;1;104:5225-37
2) DEG analysis: there is a repetition of “DEGs DEGs”
3) CMS hub genes: Word missing “To examine the CRC relevance… compiled from THE NCBI”
4) The quality of the figures needs to be improved.
5) Figure 1 should read Euler, not Eular
