# Supplementary material for: Consensus molecular subtype differences linking colon adenocarcinoma and obesity revealed by a cohort transcriptomic analysis
Source: PLoS One. 2022 May 13;17(5):e0268436. doi: 10.1371/journal.pone.0268436 (PMC9106217; doi:10.1371/journal.pone.0268436)
Supplement: Attachment — Submitted filename: Greene et al 2022_PLOS_ONE_obesity and CMS.docx [file pone.0268436.s016.docx]

Review of Greene et al

The authors have completed a series of additional analyses to strengthen the manuscript and justify their conclusions. There are unfortunately still some unclear aspects that need to be evaluated and discussed.

1) While reviewer 3 commented on the need to be more cautious in interpretation and refer to “associations” rather than effects in this cross-sectional analysis, there are still numerous sentences and the main conclusions that discuss “effects”. E.g., the conclusions start way too strong: “We conclude that obesity has CMS-specific effects in colon cancer”.
2) This is a first, preliminary analysis and not everything can be perfect. But please interpret the findings as such, with more caution, throughout the manuscript.
3) Reviewer 3 comment 3. Please provide the results with and without Asians side by side to enable the comparison. It seems like there is not corresponding figure for the overweight to normal among the full sample set.
4) Comment 3 is critical to understand the impact of race/ethnicity on study results, due to substantial possible confounding. Please acknowledge this limitation also in the discussion.
5) Comment 6 from reviewer 3 was either not understood or not done. Please add analyses removing stage IV, if adjustment is not possible.
6) Reviewer 1 comment 3: The response starting with “The normal vs cancer analysis…” is meaningless and not relevant to the paper.
7) Please understand that CMS1 is most likely familial disease and patients are significantly younger. This needs to be added as a limitation, along with the racial composition.

Despite these shortcomings of the study I remain enthusiastic of this publication. Please simply be mindful of the many limitations in the work.
