# Supplementary material for: Consensus molecular subtype differences linking colon adenocarcinoma and obesity revealed by a cohort transcriptomic analysis
Source: PLoS One. 2022 May 13;17(5):e0268436. doi: 10.1371/journal.pone.0268436 (PMC9106217; doi:10.1371/journal.pone.0268436)
Supplement: Attachment — Submitted filename: Response to reviewers.docx [file pone.0268436.s017.docx]

Review of Greene et al

The authors have completed a series of additional analyses to strengthen the manuscript and justify their conclusions. There are unfortunately still some unclear aspects that need to be evaluated and discussed.

We thank the reviewer for their helpful comments that have led to an improved manuscript. Our response to each comment is written in a blue font. Changes to the manuscript text are written in red font below.

1) While reviewer 3 commented on the need to be more cautious in interpretation and refer to “associations” rather than effects in this cross-sectional analysis, there are still numerous sentences and the main conclusions that discuss “effects”. E.g., the conclusions start way too strong: “We conclude that obesity has CMS-specific effects in colon cancer”.

We have gone through the manuscript and have identified three instances where “effects” were stated. All three have been revised to “associations”: once in the Results section of the Abstract, and twice in the Conclusions.

2) This is a first, preliminary analysis and not everything can be perfect. But please interpret the findings as such, with more caution, throughout the manuscript.

To emphasis the preliminary nature of the findings we have added text to the conclusions in the Abstract: Our findings support that obesity impacts the CRC tumor transcriptome in a CMS-specific manner. The possible associations reported here are preliminary and will require validation using in vitro and animal models to examine the CMS-dependence of the genes and pathways. Once validated the obesity-linked genes and pathways may represent new therapeutic targets to treat colon cancer in a CMS-dependent manner.

We have also added “preliminary findings” to seven paragraphs in the results section where findings have been summarized.

3) Reviewer 3 comment 3. Please provide the results with and without Asians side by side to enable the comparison. It seems like there is not corresponding figure for the overweight to normal among the full sample set.

We have performed the overweight to normal analysis for the cohort minus Asians. The new data is now shown side-by-side with the GSEA findings from the full cohort (Supplementary Figure 5). To describe the new data we have added the following text to the Results section: A sensitivity analysis was performed to determine whether inclusion of Asian patients, which were all categorized as normal BMI, affected gene set enrichment analysis comparing RNA seq data from obese to normal and overweight to normal BMI patients for each CMS. As shown in Supplemental Figure 4B, the enrichment of Hallmark gene sets in obese patients mirrored the findings observed with whole patient population (Fig. 4A). All 27 enriched gene sets in the whole population were enriched in the sensitivity analysis. However, the exclusion of Asians in the sensitivity analysis did result in significant enrichment of three EMT gene sets in obese CMS4 tumors that did not reach significance in the whole patient population. Taken together, these preliminary findings indicate CMS specific differences in obesity-regulated immune, metabolic, and stromal signaling gene set enrichment.

In addition to examining obesity-related transcriptomic differences, we examined transcriptomic differences between overweight vs. normal BMI patients for each CMS (Suppl. Fig 5A). Hallmark gene set enrichment with an FDR (p < 0.05) revealed immune-related gene set enrichment in CMS2 and CMS4. In contrast, gene set enrichment in CMS1 was strongly related to metabolic processes (heme metabolism, fatty acid metabolism, and bile acid metabolism). The only significant enrichment in CMS3 was a metabolic process gene set (oxidative phosphorylation). In a sensitivity analysis for the overweight to normal BMI comparison, we observed that all 21 enriched gene sets from the whole patient population were also significantly enriched when the population excluded Asians (Suppl. Fig. 5B). However, there were an additional 33 significantly enriched gene sets primary in Metabolism related gene sets (20 newly enriched) and Cell Cycle related gene set (9 newly enriched). In addition, 18 of the significantly enriched gene sets were observed in CMS3 tumors. These preliminary findings indicate that racial/ethnic differences may strongly influence transcriptomic differences in tumors from overweight patients.

4) Comment 3 is critical to understand the impact of race/ethnicity on study results, due to substantial possible confounding. Please acknowledge this limitation also in the discussion.

We have added the following to the limitations section: A limitation of the current study is that it was only performed in the TCGA-COAD cohort which lacked racial diversity. The impact of race/ethnicity may have led to possible confounding. CMS categorization of CRC patients across the BMI categories led …

5) Comment 6 from reviewer 3 was either not understood or not done. Please add analyses removing stage IV, if adjustment is not possible.

We agree with the reviewer that weight loss is observed in a percentage of stage IV colon cancer patients prior to diagnosis. However, as reported by Walter et al (2016) and van Zutphen et al (2019), weight loss prior to diagnosis is also observed in stage I, II, and III CRC. According to Walter et al (2016), weight loss prior to diagnosis is more common in Stage II and III CRC than in stage IV. Therefore, removing stage IV colon cancer patients would not address the issue of weight loss prior to diagnosis. The lack of data on weight loss prior to diagnosis in our study is a limitation for all colon cancer stages. Therefore, we have revised the limitations in the Discussion section in the following manner to address this point: Additional limitations of the current study are that: 1) confounding factors that are risk factors for CRC such as smoking and alcohol consumption were not assessed; 2) weight loss prior to diagnosis which has been reported in all four colon cancer stages (93, 94), was not assessed; and 3) our findings in CMS1 tumors should be interpreted with caution because CMS1 is associated with familial disease and a younger patient population.

Walter V, Jansen L, Hoffmeister M, Ulrich A, Roth W, Bläker H, et al. Prognostic relevance of prediagnostic weight loss and overweight at diagnosis in patients with colorectal cancer. The American journal of clinical nutrition. 2016;104(4):1110-20.

van Zutphen M, Geelen A, Boshuizen HC, Winkels RM, Geijsen AJ, Wesselink E, et al. Pre-to-post diagnosis weight trajectories in colorectal cancer patients with non-metastatic disease. Supportive Care in Cancer. 2019;27(4):1541-9.

6) Reviewer 1 comment 3: The response starting with “The normal vs cancer analysis…” is meaningless and not relevant to the paper.

We apologize for the lack of relevance in the first sentence of the response. The first sentence in the response was not incorporated into the manuscript. So, there was nothing to edit in the current revision.

7) Please understand that CMS1 is most likely familial disease and patients are significantly younger. This needs to be added as a limitation, along with the racial composition.

We have revised the limitations to address the comment in following manner: Additional limitations of the current study are that confounding factors that are risk factors for CRC such as smoking and alcohol consumption were not assessed; weight loss prior to diagnosis which has been reported in all four stages (93, 94), was not assessed; and our findings in CMS1 tumors should be interpreted with caution because CMS1 is associated with familial disease and a younger patient population.

Despite these shortcomings of the study I remain enthusiastic of this publication. Please simply be mindful of the many limitations in the work.
